# Computer Vision for Detection of Body Posture and Behavior of Red Foxes

**DOI:** 10.3390/ani12030233

**Published:** 2022-01-19

**Authors:** Anne K. Schütz, E. Tobias Krause, Mareike Fischer, Thomas Müller, Conrad M. Freuling, Franz J. Conraths, Timo Homeier-Bachmann, Hartmut H. K. Lentz

**Affiliations:** 1 Friedrich-Loeffler-Institut (FLI), Federal Research Institute for Animal Health, Institute of Epidemiology, Südufer 10, 17493 Greifswald-Insel Riems, Germany; Franz.Conraths@fli.de (F.J.C.); Timo.Homeier@fli.de (T.H.-B.); Hartmut.Lentz@fli.de (H.H.K.L.); 2 Friedrich-Loeffler-Institut, Federal Research Institute for Animal Health, Institute of Animal Welfare and Animal Husbandry, Dörnbergstr. 25/27, 29223 Celle, Germany; Tobias.Krause@fli.de; 3 Institute of Mathematics and Computer Science, University of Greifswald, Walther-Rathenau-Straße 47, 17487 Greifswald, Germany; mareike.fischer@uni-greifswald.de; 4 Friedrich-Loeffler-Institut, Federal Research Institute for Animal Health, Institute of Molecular Virology and Cell Biology, Südufer 10, 17493 Greifswald-Insel Riems, Germany; Thomas.Mueller@fli.de; 5 Friedrich-Loeffler-Institut, Federal Research Institute for Animal Health, Südufer 10, 17493 Greifswald-Insel Riems, Germany; Conrad.Freuling@fli.de

**Keywords:** YOLOv4, computer vision, animal monitoring, animal behavior, animal activity, animal welfare, body posture

## Abstract

**Simple Summary:**

Monitoring animal behavior provides an indicator of their health and welfare. For this purpose, video surveillance is an important method to get an unbiased insight into behavior, as animals often show different behavior in the presence of humans. However, manual analysis of video data is costly and time-consuming. For this reason, we present a method for automated analysis using computer vision—a method for teaching the computer to see like a human. In this study, we use computer vision to detect red foxes and their body posture (lying, sitting, or standing). With this data we are able to monitor the animals, determine their activity, and identify their behavior.

**Abstract:**

The behavior of animals is related to their health and welfare status. The latter plays a particular role in animal experiments, where continuous monitoring is essential for animal welfare. In this study, we focus on red foxes in an experimental setting and study their behavior. Although animal behavior is a complex concept, it can be described as a combination of body posture and activity. To measure body posture and activity, video monitoring can be used as a non-invasive and cost-efficient tool. While it is possible to analyze the video data resulting from the experiment manually, this method is time consuming and costly. We therefore use computer vision to detect and track the animals over several days. The detector is based on a neural network architecture. It is trained to detect red foxes and their body postures, i.e., ‘lying’, ‘sitting’, and ‘standing’. The trained algorithm has a mean average precision of 99.91%. The combination of activity and posture results in nearly continuous monitoring of animal behavior. Furthermore, the detector is suitable for real-time evaluation. In conclusion, evaluating the behavior of foxes in an experimental setting using computer vision is a powerful tool for cost-efficient real-time monitoring.

## 1. Introduction

Animal welfare is becoming increasingly important in animal experimentation and husbandry, and is often defined by the Five Freedoms concept [1], the Five Domains concept, or the complementary use of both [2,3]. Thus, due to its multidimensional character, it is influenced by many factors [4]. For its detection based on animal movements, different approaches have been applied in recent years. Séneque et al. [5] found that altered welfare in horses was associated with their body postures. Besides active motion, sleeping behavior can be used as one indicator of animal welfare [6]. Furthermore, the monitoring of animal activities has been used to draw conclusions on animal welfare [7]. In particular, changes of behavioral activity can provide information about the welfare or disease status of an animal [8,9,10]. Observation, measurement, and evaluation of animal behavior provide important indicators for the determination of animal welfare [11]. Furthermore, animal behavior is often associated with certain postures and locomotion [12]. Fureix et al. [13] used horse postures (analyzed by geometric morphometrics) to characterize behavioral categories. Animal behavior is an important manifestation, which can be linked to the health and welfare status of animals. Therefore, conclusions about animal health and welfare can be drawn by tracking animals and detecting their posture and activity.

For monitoring, unbiased video observation of animal behavior is particularly suitable, since the presence of humans may change or influence the behavior of animals [9,14,15,16]. Moreover, manual observation has a number of disadvantages. It is time consuming, costly, and unsuitable for larger animal populations [17]. More importantly, the accuracy of the manual observation depends on the observer’s experience and judgment [17], which may lead to observer bias [18].

An automated system may help to overcome at least some of the above-mentioned limitations and may be used to detect behavioral changes, e.g., unusual behavior caused by disease. Moreover, such a system could automatically alert laboratory personnel in case of unusual behavior and thus support animal welfare, health, and animal management [12]. Furthermore, automatic monitoring methods can be useful for continuous monitoring and detection of events [19] such as an animal caretaker in the room.

The application of sensors such as accelerometers or RFID (radio-frequency identification) chips is one way of measuring activity or locomotion. Data obtained using accelerometers, usually attached to the animals as leg sensors, have been used to classify cattle activities (e.g., walking, lying, standing) [20]. Kaler et al. [21] used data from accelerometer and gyroscope sensors to detect lameness in sheep. Furthermore, Diosdado et al. [22] used the accelerometer data for the classification of behaviors in dairy cows, i.e., feeding, standing, and lying. For the application of the RFID technology, chips can be either implanted or attached to collars, ear tags, or anklets [23]. There have been attempts for implementing automatic monitoring of animals using RFID technology [24,25]. However, the use of RFID is always associated with certain invasiveness of the animals, since the sensors (RFID chips) must be attached to the animals or implanted. In particular, the implantation may cause stress for the animals [26] and can thus affect their subsequent behavior. The application of video surveillance has the advantage that no sensors or tags need to be placed in or on animals, which avoids stress.

Computer vision based techniques are objective, contact-less, and low-cost methods. They offer an important basic tool in the study and monitoring [27] of animal behavior. Nasirahmadi et al. [28] tested three detector methods (region-based fully convolutional network, single shot multibox detector, and faster regions with convolutional neural network) for the detection of lying and not lying pigs. Yang et al. [29] applied an automatic recognition framework based on a fully convolutional network to detect the daily behavior of sows by analyzing motion (to detect movement, medium active, or inactive behavior) and image analyses (to detect drinking, feeding, and nursing). Another effective real-time object detection algorithm is YOLO (You Only Look Once) [30]. Wang et al. [31] used YOLOv3 (You Only Look Once version 3) to detect six different categories of behavior (e.g., drink, feed, stand) in group-housed hens in a self-breeding system and used the frequency of mating as a welfare indicator for the group. YOLOv4 was used by Jiang et al. [32] to detect goats and to recognize the behavior of group-housed goats (eating and drinking by position of the goats and active/inactive based on the movements). In addition, YOLOv4 has been applied to detect and monitor the motion and activity levels of red foxes [33], but without identification of specific behaviors.

The automated deduction of animal behavior patterns by a combined evaluation of the detection of body postures and the determination of activity levels is missing so far. For this purpose, we demonstrate the application of deep learning for the detection, tracking, activity, and behavior determination of red foxes (*Vulpes vulpes*) during an experimental study, which was being conducted to measure the long-time immunogenicity and efficacy of an oral rabies vaccine in these animals [34]. To this end, we used video surveillance data of the foxes generated as part of this experimental study. In particular, a convolutional neural network (CNN) (YOLOv4) is trained for red fox body posture detection, e.g., ‘standing’, ‘lying’, and ‘sitting’. The results of this detection can be used to infer different activity levels [33], which can then be used in combined evaluation with the detected posture to determine different behavior. The presented technique can be applied to detect posture patterns of animals, including behavior determination.

## 2. Materials and Methods

### 2.1. Experimental Setup

The animals considered here are red foxes (*Vulpes vulpes*) of the fur color variant ‘silver fox’ [35]. The experimental study with 23 foxes was conducted over 450 days at the Friedrich-Loeffler-Institut (FLI), Greifswald, Insel Riems, Germany [34]. During that time the foxes were separately kept in cages, sized 3.18 m × 1.4 m × 1.75 m (length × width × height), equipped with a platform, 0.92 m × 1.4 m (length × width) at height of 0.8 m above the bottom of the cage. In addition, each cage was equipped with a hut. In the study, a novel oral vaccination regime against rabies was tested. This required animal blood to be sampled in regular intervals. To this end, the foxes were anesthetized [34]. All invasive procedures, i.e., blood sampling, infection, transponder application, euthanasia, were conducted under anesthesia by applying 0.5–1 mL of Zoletil (Virbac, France; 1 mL contains 50 mg Tiletamin, and 50 mg Zolazepam). It takes 7 to 10 min before you can start with the manipulations. Zoletil does not require an antidote, because it is a short time anesthesia. The increased activity after anesthesia may be reflective of the recovery phase. It is known that in canids, the half-life of tiletamine is 1.2 h and that of zolazepam is 1 h. During the recovery phase of canids from anesthesia with Zoletil^®^, tiletamine therefore has an even longer effect than zolazepam and excitation states and increased movement can occur [36]. Housing and maintenance of the animals complied with national and European legislation and guidelines for the veterinary care of laboratory animals [37]. Food and water were provided according to the species-specific requirements and were also individually adjusted. The foxes received enrichment (such as ball, kong) at irregular intervals to improve animal welfare, which were not considered in our study. The animals were visually inspected daily and the cages cleaned on a regular basis. The availability of external monitoring, including recording by video cameras, was one of the requirements for approval. Therefore, every fox was monitored via two cameras (ABUS IR HD TVIP61500, ABUS, Wetter, Germany). Each cage was equipped with two cameras hung on the opposite narrow sides of the cage. Together, 33 TB of video data was recorded discontinuously (due to memory requirements), on 73 different days. We have used image data that was extracted from video data of all foxes for training the algorithm. For the exemplary application shown below, we restrict ourselves to analyzing video data of 1 single red fox on 6 different days, spanning a period of 11 days. Table 1 shows the evaluated video data and the times of events such as anesthesia or cage cleaning.

On day 3, anesthesia is administered at 09:56, the red fox shows normal behavior until this time, then lies for about 1 h followed by a wake-up phase (approximately 11:00 to 11:30) in which the red fox repeatedly crawls a bit, then repeatedly starts to stand up, walks a few steps and sat down or lies down again. Then there follows a phase in which the red fox very excitedly walks around a lot in the cage; this lasts until about 14:15. From then on the sitting and lying times between the walking become longer and longer until the red fox lies down at 16:01 and sleeps until 17:08.

### 2.2. Ethical Approval

The animal experiment was authorized by the local authority in Mecklenburg-Western Pomerania (Landesamt für Landwirtschaft, Lebensmittelsicherheit und Fischerei Mecklenburg-Vorpommern, # FLI-7221.3-1-087/16) and conducted in accordance with national and European legislation and guidelines for the veterinary care of laboratory animals [37].

### 2.3. Image and Video Data

The resolution of the video data was 1280 pixels (horizontal) × 720 pixels (vertical) and had a frame rate of 15 frames per second (fps). An image set was created by extracting single frames from the video data. For the image set, videos of all 23 red foxes with different body postures were used. No adjacent frames were extracted and frames with different illumination conditions (night and day) were used. The image set consisted of 8913 images. The red fox on each image of the image set was manually labeled using the software LabelImg [38], and attributed to one of the three classes ‘sitting’, ‘standing’, and ‘lying’. Lying was defined as lying prone, lying on the side, lying curled up, or lying on the back. Standing was defined as a quadruped position. Sitting was regarded as an intermediate posture, i.e., the two hind legs not righted and the two forelegs righted on the floor.

The image set was split into a training (80%—7129 frames) and a test set (20%—1784 frames) (Table 2), maintaining the relation of the three labeled classes. The training set was used to train the YOLOv4 object detection algorithm and the test set to evaluate the trained model for the red fox posture detection.

### 2.4. Environment Configuration

The processor used in the study was an Intel Xeon E5-2667 v4 with 3.20 GHz, 377 GB Ram, and NVIDIA K80 with 2 GPUs and 24 GB video RAM. The operating system was CentOS 7. The algorithm was developed by using a Jupyter notebook [39] and Python 3.6.8 [40].

### 2.5. Automatic Evaluation: Red Fox Detection and Posture Classification

The detection of red fox postures was implemented by using the deep learning algorithm YOLOv4. The algorithm YOLO is a one-stage object detection algorithm for real-time object detection based on CNN [30,41]. In this study, we used version 4 of YOLO, which consists of a ‘head’, a ‘neck’, and a ‘backbone’ [42]. The head is used to implement the object detection [42], and is the YOLOv3 algorithm [41]. The neck is used to collect feature maps from different stages and is based on a path aggregation network (PAN) and SPP spatial pyramid pooling (SPP) [42]. The backbone is used for training and feature extraction and it is a CSPDarknet53, which is an open source neural network framework [41,42]. YOLOv4 is a state-of-the-art detector, which is faster and more accurate than other available detectors [42]. The training of the red fox posture detector based on YOLOv4 was performed with the parameters from Table 3 and the training set (Table 2).

In this study, we have limited the detectable classes for postures exclusively to three different postures, i.e., ‘sitting’, ‘lying’, and ‘standing’. A detailed description of the training procedure is given in the Appendix A.

### 2.6. Evaluation of Model Performance

In order to verify the performance of the model, the following indicators were determined:

(i) Mean average precision (mAP) (Equation (2));
(ii) Precision (Equation (3));
(iii) Recall (Equation (4));
(iv) Detection speed.

Intersection over Union (IoU) was used to determine if a detection was true positive or false positive (see Equation (1)). When IoU≥0.5, it was true positive and false positive if IoU<0.5. If an image was labeled and the model does not detect anything, it was false negative. The following values were computed:

(1) $$IoU=\frac{area\left(BB_{p}\right)\cap area\left(BB_{gt}\right)}{area\left(BB_{p}\right)\cup area\left(BB_{gt}\right)},$$

(2) $$mAP=\frac{\sum_{c=1}^{C}AP(c)}{C},$$

(3) $$precision=\frac{TP}{TP+FP},$$

(4) $$recall=\frac{TP}{TP+FN},$$

where IoU: Intersection over Union, $BB_{p}$: predicted bounding box (BB) from the model, $BB_{gt}$: ground-truth bounding box (e.g., manually labeled), AP: average precision, *C*: number of classes, TP: number of true positives, FP: number of false positives, FN: number of false negatives. The AP is a measure for the detection accuracy of the model (for more details see [33,43]). Here we used the 11-point interpolated AP [43]:

(5) $$AP=\frac{1}{11}\sum_{r\in \{0,0.1,\cdots ,1\}}p_{interp}\left(r\right)$$

with

(6) $$p_{interp}\left(r\right)=\max_{\hat{r}:\hat{r}\geq r}p\left(\hat{r}\right),$$

where $p(\hat{r})$ is the precision at recall $\hat{r}$. Equation (6) results in a smoothening of the precision-recall curve.

For each image *f*, the trained object detection algorithm returns whether a red fox is on the image, and if so, the class (‘lying’, ‘sitting’, ‘standing’), the confidence of the detection, and the bounding box center position ($x_{f}$, $y_{f}$), and width and height—standardized between 0 and 1, respectively.

### 2.7. Automatic Evaluation: Activity Analysis

The bounding boxes were used to measure the activity level of the red fox [33]. To this end, the movement of the center of the bounding box between two consecutive frames was determined. This movement of the center of the BB corresponds to the distance covered by the red fox between two consecutive frames. $m_{f,f+1}=\sqrt{{\left(x_{f+1}-x_{f}\right)}^{2}+{\left(y_{f+1}-y_{f}\right)}^{2}}$ with $\left(x_{f},y_{f}\right)$: coordinates of the center of the BB in frame *f* and $\left(x_{f+1},y_{f+1}\right)$: coordinates of the center of the BB in frame f+1. For the calculation of the mean vector norm, a sliding window of 5 s with a step size of 1 s was used. ${\bar{m}}_{t}=1/F\sum_{f=1}^{F-1}m_{f,f+1}$ with mean vector norm ${\bar{m}}_{t}$, time period *t*, and number of frames *F* in *t*. The maximum of the mean vector norm for different periods and kinds of movement behavior can be used to determine thresholds for different activity levels. Three activity levels were considered:

(i) Highly active: considerable movement of the bounding box (BB), i.e., the localization of the red fox changes, e.g., walking or running;
(ii) Active: slight movement of the BB, i.e., the localization of the red fox does not change, but there is some movement inside the BB, e.g., rotation or minimal movements, such as scratching or stretching;
(iii) Inactive: no movement of the BB, i.e., the red fox does not move, e.g., lying, sitting, or standing still.

To distinguish between the activity levels, the thresholds from Schütz et al. [33] were used (mean norm ≥0.0094: highly active; 0.0054 to 0.0094: active; 0 to 0.0054: inactive).

For all three postures (lying, sitting, standing), highly active, active, and inactive variants are possible.

### 2.8. Automatic Evaluation: Behavior Analysis

For behavioral analysis the activity levels were considered along with body posture to draw conclusions about the behavior of the respective red fox. Therefore, a behavior was assigned to each possible combination of a body posture and activity level (see below).

### 2.9. Workflow for Automated Video Evaluation

For the evaluation of the videos we used the trained red fox detector as described above. The video analysis was implemented as follows:

1. Frame extraction (5 frames per second);
2. Red fox posture detection on each frame;
3. Activity analysis using the BB values for the activity level determination;
4. Behavior analysis using the posture and activity level for the behavior classification.

**Figure 1:**
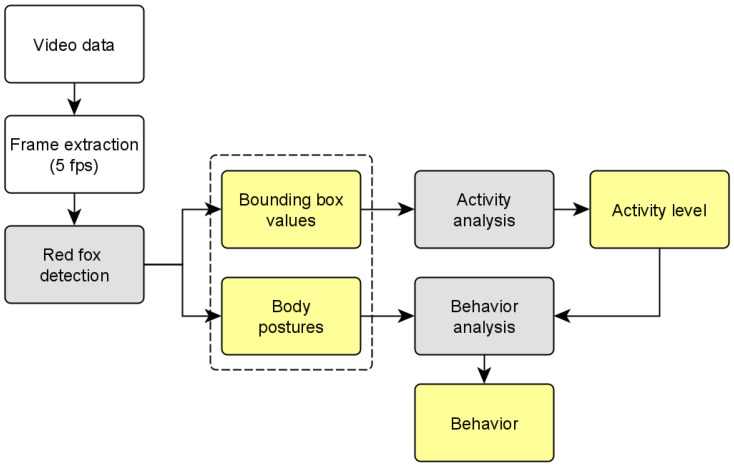
Workflow: video data evaluation. White: raw video material. Gray: detection and analysis. Yellow: results.

The evaluation workflow is depicted in Figure 1.

For the joint evaluation of both cameras of the same animal, each video was evaluated separately for the same period. It is possible that the evaluation of both cameras differs, e.g., if the fox is not completely visible for camera 1 because the legs are in the blind spot, the fox is classified as lying for camera 1, while camera 2 (seeing the complete fox) classifies it as standing. In case of mismatches between the cameras, the larger vector norm and the body posture with the higher confidence was chosen. To avoid single individual false classifications for the body posture, a sliding window of 5 s duration was used to select the most frequently occurring body posture.

## 3. Results

### 3.1. Model Training and Evaluation

The performance of the model was evaluated by comparing the label of manually labeled images (test set) with the results of the automated detection of the trained model. The results for the three classes are shown in Table 4, and the overall performance is shown in Table 5.

The precision and recall of the model are 98.61% and 95.12%, respectively; the average IoU is 0.91, the mAP is 99.91% and the detection speed reaches 73.31 ms per frame.

**Figure 2:**
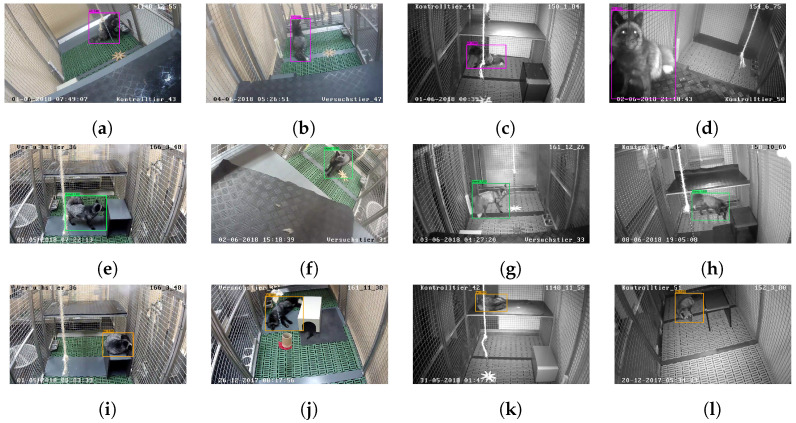
Detection of red foxes in single frames. (**a**–**d**) detection examples of sitting foxes; (**e**–**h**), standing foxes; and (**i**–**l**) lying foxes. Two day scene images (left) and night scene images (right), respectively.

Figure 2 shows examples of red fox detection for each of the three postures ‘sitting’ (Figure 2a–d), ‘standing’ (Figure 2e–h), and ‘lying’ (Figure 2i–l) for day and night scenes.

#### 3.1.1. Activity Detection

**Figure 3:**
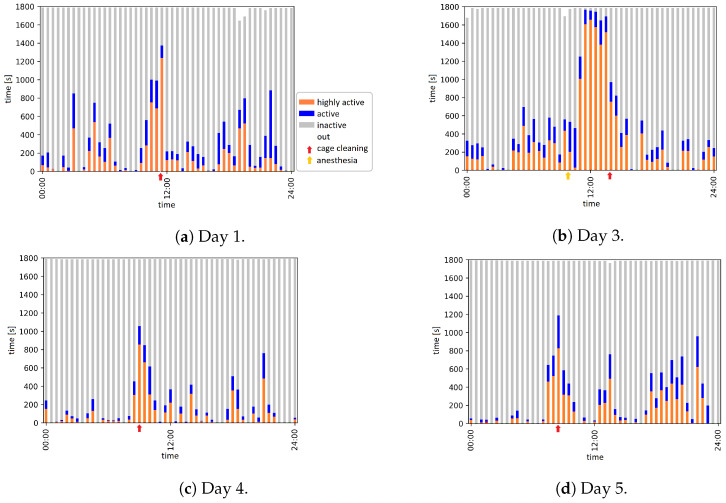

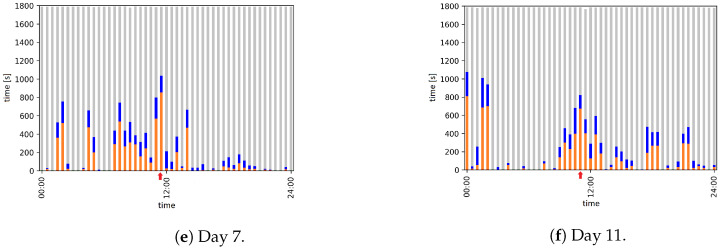
Activity overview of six days from a time period of 11 days.

Figure 3 provides overviews of the activity level for the evaluated days during the 11-day observation period.

For example, day 1 represents the normal activity of the red fox. More active and inactive phases alternate throughout the day. The period during which the animal caretakers entered the room and cleaned the cages related with increased red fox activity. This can be seen particularly well on day 1, from 11:30 to 12:00, where the largest amount of ‘highly active’ coincides with the presence of an animal caretaker in the room (11:30 to 11:55). The anesthetic phase, i.e., anesthesia and subsequent recovery phase, on day 3 relates with the active phase on that day. Activity increased suddenly with the beginning of the wake-up phase at 11:00. From 11:00 to 14:00, the activity level was almost only ‘highly active’ and related with walking around in the cage after the wake-up phase. From 14:00 to 16:00, the proportion of ‘highly active’ decreased and the red fox showed the activity level ‘inactive’ for a longer time. This is followed by a complete hour (16:00 to 17:00) in the activity level ‘inactive’, which coincided with the sleeping phase from 16:01 to 17:09.

#### 3.1.2. Posture Detection

**Figure 4:**
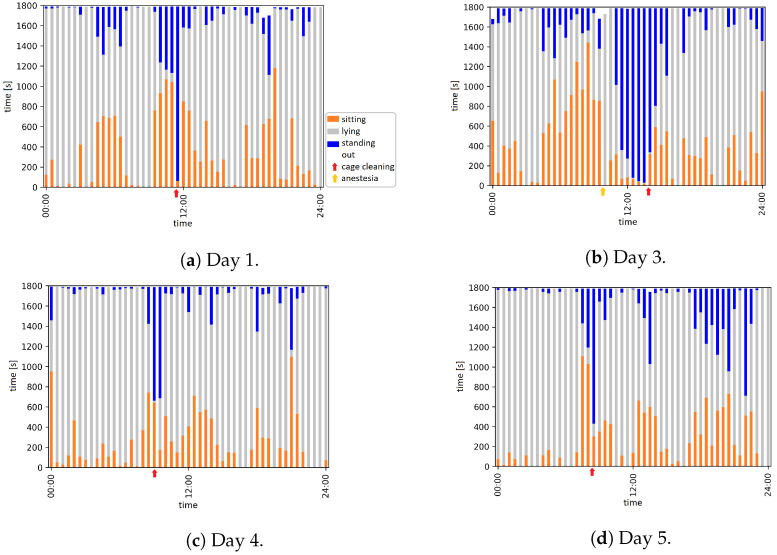

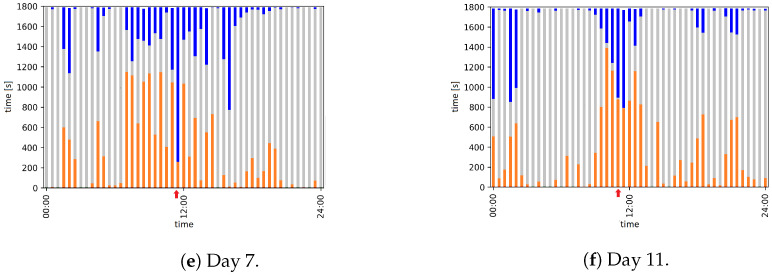
Posture overview of 6 days from a period of 11 days.

The trained model was used to classify the postures, ‘sitting’, ‘lying’, and ‘standing’. Figure 4 shows the posture overviews for all recorded days during the observation period of 11 days.

In all plot, periods when an animal caretaker was in the room were very well recognizable, e.g., from 11:30 to 11:55 on day 1, (Figure 4a), and also on day 7 (Figure 4e). The red fox was only standing or sitting, but hardly lying. The anesthesia on day 3 with the subsequent wake-up phase (described in Section 3.1.1) is also reflected in the classified postures. On day 11, there was a phase in which the red fox almost exclusively showed the postures ‘sitting’ and ‘standing’, which lasted from about 10:00 to 12:00. The classified postures agree with the manually analyzed video data for this period, i.e., the red fox mostly sat or stood from 10:00 onwards, it laid down at 12:20. Another remarkable half hour was on day 5 from 9:00 p.m. to 9:30 p.m. Here the body posture detection shows that the red fox only used the body postures ‘sitting’ and ‘standing’. The classified postures are consistent with the video.

**Figure 5:**
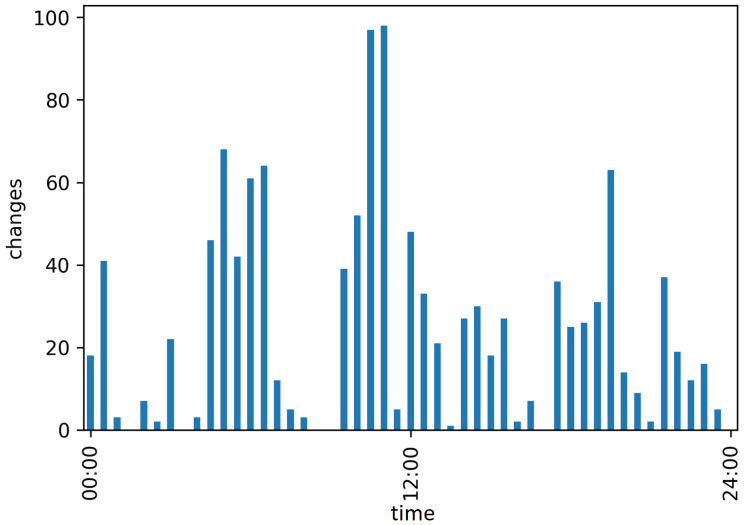
Number of changes on day 1 in half hour steps.

The classified postures were used to determine the numbers of body posture changes. The numbers of changes for day 1, summed up per half hour, are shown in Figure 5.

**Figure 6:**
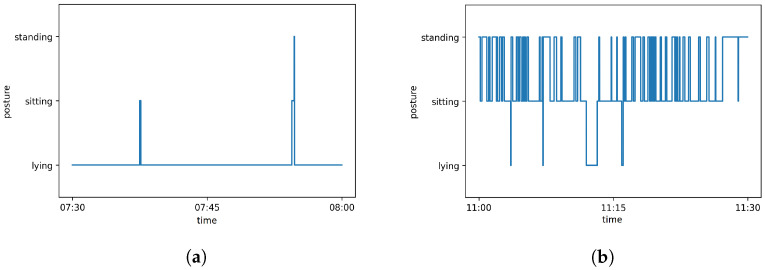
Postures of the red fox over 30 min during two different periods. Each vertical line represents a posture change. (**a**) Day 1—07:30 to 08:00; (**b**) Day 1—11:00 to 11:30.

Randomly three arbitrarily selected half-hour videos were manually analyzed and compared with the determined number of changes. For this purpose, one video with many changes (11:00 to 11:30—98 changes), one with few changes (07:30 to 08:00—5 changes), and one with no changes (17:00 to 17:30—zero changes) were randomly selected from all the videos of day 1. In all three videos, the automatic detected numbers of changes matched the numbers seen during manual inspection of the video and, in addition, the time points of the changes matched. Figure 6 shows a timeline for each of the periods with posture changes.

The five changes during the period lasting from 07:30 to 08:00 period are illustrated in Figure 6a, where each vertical line represents a posture change. A second period (Figure 6b) depicts all 98 posture changes that were recorded between 11:00 and 11:30. Most of the changes occurred between the postures ‘sitting’ and ‘standing’.

#### 3.1.3. Behavior Detection

**Figure 7:**
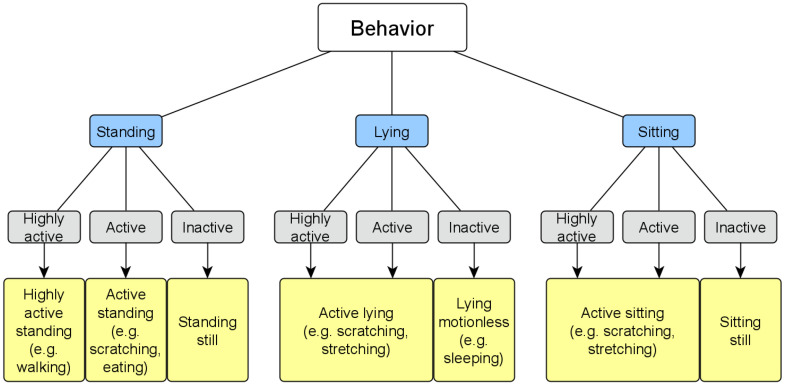
Decision tree: Determination of behavior (yellow) through a combined analysis of body posture (blue) and activity level (gray). For example, if a fox is classified as ‘active’ and ‘standing’, it shows the behavior ‘active standing’. This refers to a standing fox with an activity level of ‘active’, and this could be scratching, eating, etc.

To determine the behavior, all combinations of posture (’lying’, ‘sitting’, and ‘standing’) and activity level (’inactive’, ‘active’, and ‘highly active’) were considered and a behavior assigned to each combination, i.e., ‘highly active standing’, ‘active standing’, ‘standing still’, ‘active lying’, ‘lying motionless’, ‘active sitting’, and ‘sitting still’. This is shown as a decision tree in Figure 7.

With the decision tree (Figure 7) it is possible to generate a continuous behavior overview with a resolution of one value per second (see Section 2.7). For example, if a red fox is classified as ‘standing’ in an image, the combined view with the determined activity level provides the behavior of the fox. If the activity level is ‘highly active’, the behavior of ‘highly active standing’ can be inferred. If the corresponding activity level is ‘active’, this means that the fox is ‘standing active’. The last possible combination is the activity level ‘inactive’, which means that the behavior of the fox is ‘standing still’.

**Figure 8:**
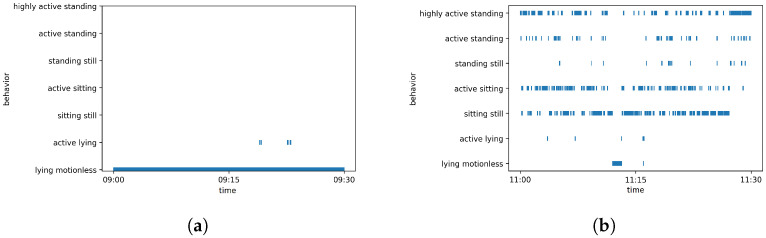
Overview of the exhibited behavior of the red fox in timelines of 30 min for two different periods. (**a**) Day 1—09:00 to 09:30; (**b**) Day 1—11:00 to 11:30. Note: The fox does not show two behaviors at the same time at any point, although it may appear like that in the figure due to the high density of data points.

The determined behavior of the red fox is exemplarily represented by two timelines over half an hour each in Figure 8.

The first half hour (Figure 8a) shows the behavior of a sleeping fox. It can be seen that the animal was ‘lying motionless’ for almost the entire observation period. Only at two short moments was there movement during sleep. The behavior coincides with the real behavior as determined by visual inspection of the video data, where it became evident that the fox slept curled up and changed its lying position only twice. In the second timeline (Figure 8b), one can see that the red fox showed all the defined behaviors. Here, visual inspection of the video showed that the red fox changed its location very often, and changed frequently between the body postures ‘sitting’, ‘standing’, and ‘lying’.

## 4. Discussion

We were able to train a classifier with a very high precision for posture detection, and we can determine animal behavior in detail in a high temporal resolution. Our results suggest that the presented method may be useful for monitoring animals, especially posture, behavior, activity, and additional posture change monitoring. In our setting, the results show that the model achieves high performance for the posture detection. Moreover, the detection speed is sufficient for a real-time detection with 5 fps, which was used in this study. Furthermore, the animals do not need to be equipped with a sensor or collar, as was the case in other studies (e.g., [20,44]). This non-invasive approach is a major advantage of computer vision [45].

Activity determination works in the same way with the new method for the posture detection in our previous study on red fox detection [33]. The activity overview can also be used to detect events like the anesthesia or the presence of an animal caretaker in the room. In particular, increased activity in the presence of humans illustrates their influence on animal behavior and the advantages of video observation in getting an—at least in this respect—unbiased insight. Changes in movement patterns can provide information relevant to animal welfare and health [44,46], especially over long terms these data can be achieved automatically by the proposed approach.

In this study, three major postures of red foxes were investigated; ‘lying’, ‘sitting’, and ‘standing’. The posture ‘lying’ consists of all the shown lying postures of the red fox, including ‘lying curled up’, ‘lying on side’, ‘lying on back’, and ‘lying prone’. The red fox also shows other body postures like ‘standing on the hind legs’ or ‘standing front end lowered’. For these rare postures, it is difficult to create a large enough image set for training, e.g., only 25 images could be labeled with the posture ‘standing front end lowered’ from the total extracted image set. The classified postures are also suitable for determining periods with special events. Furthermore, in contrast to pure activity monitoring, periods with an unremarkable activity, but a noticeable ratio of the postures were detected. For example, in the period from 09:00 to 09:30 on day 4, the red fox shows almost only ‘sitting’ and ‘standing’ postures (Figure 4c), but no particularly striking values regarding of activity levels. Thus, the different duration of the postures could be used as an indicator of animal behavior. As an example, a reduction of lying time is a typical behavior change before a calving in dairy cows [47]. Similarly, the determination of the number of changes is an indicator for behavioral changes. In dairy cows, an increase of posture changes is another typical behavior change before calving [48].

Analysis of the behavior as a result of the combined evaluation of activity and posture yields a detailed overview of the behaviors under consideration, i.e., ‘highly active standing’, ‘active standing’, ‘standing still’, ‘active lying’, ‘lying motionless’, ‘active sitting’, and ‘sitting still’. The use of the developed decision tree provided good insights into detailed behavior. Even though we did not provide a detailed paired analysis here, given the high accuracy of the used algorithm (as discussed in Section 3.1), the method is capable of reproducing the manual results. However, different kinds of behavior that show the same combination of activity level and posture cannot be distinguished. For example, the behaviors ‘lying motionless—sleeping’ and ‘lying motionless—awake’ have the same combination and the decision tree provides ‘lying motionless’. However, sleeping and resting phases could be used to refine these categories, which may be relevant as indicators of animal welfare [6]. There is a further limitation in assessing welfare based on behavior, e.g., ‘highly active standing’ could be due to the fox’s interaction with enrichment (indicating good welfare) and also pacing (indicating poor welfare). Thus, the number of determinable behaviors is a limitation [45]. This limitation can be minimized, for example, by training a model with more postures.

Our proposed method is based on single snapshots—as opposed to estimating behavior from long-time windows. It shows a high precision and accuracy here (correct detection of behavior compared to manual observation). In order to estimate the accuracy of long-term evaluations, a long-term study using a large amount of video data with this method might be part of future research.

We showed that computer vision systems are useful to generate activity, posture, and behavior overviews and the number of posture changes.

## 5. Conclusions

The aim of this study was to investigate the potential of computer vision for the detection of red foxes and the classification of their body postures to use the results for activity, posture, and behavior analysis. On the basis of the YOLOv4 algorithm, a model for the detection of red foxes was realized that classifies the body postures ‘lying’, ‘sitting’, and ‘standing’. Along with the subsequent analysis of the posture changes, activity levels, and behavior of the red foxes, this study provides a method for seamless monitoring.

The generated daily, weekly, or monthly overviews (activity, posture, behavior) can be used to monitor animal activities, posture, and behavior, and may thus help to establish indicators for animal welfare.

## Figures and Tables

**Table 1:** Subset of days evaluated and of external events (anesthesia, cleaning). Video data does not exist for days not listed in the period and cannot be evaluated.

Day	Time	Event
1	11:30 to 11:55	Animal caretaker is in the room and cleans the cage
3	09:56	Anesthesia
	13:59 to 14:09	Animal caretaker is in the room and cleans the cage
4	09:18 to 09:31	Animal caretaker is in the room and cleans the cage
5	08:22 to 08:34	Animal caretaker is in the room and cleans the cage
7	11:30 to 11:55	Animal caretaker is in the room and cleans the cage
11	11:18 to 11:34	Animal caretaker is in the room and cleans the cage

**Table 2:** Splitting of the image set (8913 frames) into a training and a test set in the ratio 80% to 20%. Shown also is the number of frames per subset for each behavioral postures ‘lying’, ‘sitting’, and ‘standing’.

	Total	Lying	Sitting	Standing
Training set	7129 frames	775 frames	3688 frames	2370 frames
Test set	1784 frames	194 frames	922 frames	593 frames

**Table 3:** Parameters of the YOLOv4 red fox detection model.

Parameter	Value
Input size	416×416
Classes	3
Maxbatches	6000
Filters	24
Steps	4800, 5400
Learning rate	0.001
Batch size	64

**Table 4:** Performance of the trained model for the classes ‘lying’, ‘sitting’, and ‘standing’.

Class	Precision	Recall	Average IoU	AP
Sitting	99.24%	99.57%	0.93	99.97%
Lying	96.95%	98.45%	0.90	99.79%
Standing	98.16%	99.16%	0.90	99.96%

**Table 5:** Overall performance of the trained model.

Precision	Recall	Average IoU	mAP	Detection Speed
98.61%	95.12%	0.91	99.91%	73.31 ms

## Data Availability

The data sets during and/or analyzed during the current study are available from the corresponding author on reasonable request.

## Abbreviations

    The following abbreviations are used in this manuscript:

AP	average precision
BB	bounding box
CNN	convolutional neural networks
FLI	Friedrich-Loeffler-Institut
FP	false positive
FN	false negative
IoU	intersection over union
mAP	mean average precision
TP	true positive
YOLO	you only look once

## Appendix A

In this section, we provide a detailed description on how to train the algorithm. The procedure is very similar to our previous work [33]. The training was implemented following the instructions of the YOLOv4 Github page [49] with pre-trained weights (i.e., a pre-trained weights-file yolov4.conv.137, downloaded from GitHub [49]) and the parameters shown in Table 3.

The algorithm was trained as follows:

1. Download and extract YOLOv4 from GitHub [49].
2. Copy the content of cfg/yolov4-custom.cfg to the new created file yolo-obj.cfg and change the following lines:
  line 3:  batch=64 
  line 4:  subdivisions=1 
  line 8:  width=416 
  line 9:  height=416 
  line 20:  max_batches=6000  (classes∗2000)
  line 22:  steps=4800,5400  (80 and 90% of maxbatches)
  lines 603, 689, 776:  filters=24 ((classes+5)∗3)
  lines 610, 696, 783:  classes=3 
3. Create a file obj.names with the name of each object in separate lines:
  sit
  lie
  stand
4. Label each image of the image set, such that for each image there exists a .txt file with the following values for every labeled object:
  <object-class> <BB x_center> <BB y_center> <BB width> <BB hight>
  with <object-class> an integer between 0 and number of classes−1, and  <BB x_center>, <BB y_center>, <BB width> and <BB hight> are float values between (0,1], relative to the image height and width. Thus, the directory with the images contains a .txt file for each image with the same name.
  Create the files train.txt and test.txt. Split the image set into a training and test set and save the file names of the images, with respect to the full path relative to the directory darknet, in the respective file (one file name per line).
5. Create a file obj.data containing the number of classes and paths to train.txt, obj.names, and the backup folder:
  classes = 3
  train = data/train.txt
  names = data/obj.names
  backup = backup/
6. For starting the training, run the code:
  ./darknet detector train obj.data yolo-obj.cfg yolov4.conv.137
  The training can take several hours. During training the trained weights are saved in the backup/ directory, yolo-obj_xxxx.weights every 1000 iterations and yolo-obj_last.weights every 100 iterations. After training the final weight, yolo-obj_final.weights is also stored there.
7. Evaluate the results for trained weights:
  ./darknet detector map obj.data yolo-obj.cfg
  backup/yolo-obj_final.weights
8. Using the trained detector:
  ./darknet detector test obj.data yolo-obj.cfg
  backup/yolo-obj_final.weights
